# Risk Factors Associated with Cartilage Defects after Anterior Cruciate Ligament Rupture in Military Draftees

**DOI:** 10.3390/jpm12071076

**Published:** 2022-06-30

**Authors:** Ting-Yi Sun, Chun-Liang Hsu, Wei-Cheng Tseng, Tsu-Te Yeh, Guo-Shu Huang, Pei-Hung Shen

**Affiliations:** 1 Department of Orthopedic Surgery, Tri-Service General Hospital, National Defense Medical Center, Taipei 114, Taiwan; ericmaryptt@gmail.com (T.-Y.S.); windoverture@gmail.com (C.-L.H.); tsutey@gmail.com (T.-T.Y.); 2 Division of Traumatology, Department of Surgery, Tri-Service General Hospital, National Defense Medical Center, Taipei 114, Taiwan; 3 Department of Biomedical Engineering, National Yang Ming Chiao Tung University, Taipei 112, Taiwan; 4 Department of Anesthesiology, Tri-Service General Hospital, National Defense Medical Center, Taipei 114, Taiwan; ndmc_wechern@yahoo.com.tw; 5 Department of Radiology, Tri-Service General Hospital, National Defense Medical Center, Taipei 114, Taiwan; gshuang5@gmail.com

**Keywords:** anterior cruciate ligament rupture, ACLR, conservative treatment, risk factors, osteoarthritis, graft rupture, military

## Abstract

This study aimed to evaluate the different clinical results and factors associated with cartilage defects in military draftees who underwent different treatments after anterior cruciate ligament (ACL) rupture. Overall, 105 patients who had sustained ACL rupture were military draftees who underwent a conscription examination for physical status assessment from January 2012 to December 2020. Patients were divided into three groups: conservative treatment after ACL rupture, status post-anterior cruciate ligament reconstruction (ACLR), but graft rupture, and status post-ACLR with graft intact. Inter-group comparisons and statistical analyses were performed for age, body mass index (BMI), thigh circumference difference, side-to-side difference in anterior knee translation by KT-2000, meniscus tear, and cartilage defect. Multivariate logistic regression analysis was used to determine the factors associated with cartilage defects. The multivariable regression model showed that BMI (odds ratio OR: 1.303; 95% CI: 1.016–1.672; *p* = 0.037), thigh circumference difference (OR: 1.403; 95% CI: 1.003–1.084; *p* = 0.034), tear of lateral meniscus (LM) and medial meniscus (MM) (OR: 13.773; 95% CI: 1.354–140.09; *p* = 0.027), and graft rupture group (OR: 5.191; 95% CI: 1.388–19.419; *p* = 0.014) increased the risk of cartilage defects. There was no correlation between cartilage defects and age, KT-2000 difference, tear of LM or MM, or graft intact group. Progression of osteoarthritis was concerned after ACL rupture, and this study identified several factors of post-ACLR graft rupture, greater thigh circumference difference, BMI, and meniscus tear of both LM and MM affecting cartilage defects, which represent early degenerative osteoarthritis changes of the knee. The results of this study should be customized for rehabilitation and military training, especially in military draftees with ACL injuries.

## 1. Introduction

Anterior cruciate ligament rupture is one of the most common sports injuries [1], and the incidence of ACL injuries was 10-times among military service members than the general population [2]. ACL rupture is often combined with meniscal tears, articular cartilage wear, thigh muscle atrophy, and abnormal knee joint laxity [3,4,5,6]. Therefore, anterior cruciate ligament reconstruction (ACLR) is used to restore joint stability and prevent subsequent osteoarthritis (OA) [7,8,9]. However, more than 50% of military service members continue to have some degree of activity limitations following ACL reconstruction; thus, there is a significant healthcare burden, especially in high physically demanding situations [10,11,12]. Identifying these patients, in particular military service members, is essential for specific military training.

Conservative treatment remains an option for the treatment of ACL rupture, but it is still debated whether to use conservative treatment or surgical intervention. Posttraumatic OA is the most common consequence of ACL rupture [7,13,14,15,16,17]. Some studies have indicated that ACL reconstruction cannot prevent the development of OA [4,7,18], and the prevalence of OA is higher in the knee with ACLR than in non-injured knees [4,19,20]. Furthermore, graft failure after ACLR is a devastating event, and there are many factors affecting graft failure [21,22]. The sequelae of graft failure, especially OA, are still clinically challenging problems [3,23,24,25,26]. However, the cause of OA is multifactorial and long-term outcomes after ACL injury and post-ACLR are influenced by many associated factors, such as the status of the articular cartilage, meniscus, BMI, state of the quadriceps muscles, and knee joint laxity [5,27,28,29,30,31,32,33].

OA is generally regarded as a disease involving all structures in the knee, including articular cartilage, meniscus, ligaments, synovium, capsular structures, and bone marrow [34]. Cartilage defects are associated with early degenerative changes and OA development [5,34]. The condition of the ACL graft, meniscus injury, and cartilage defect can be evaluated by magnetic resonance imaging (MRI), as it has a higher resolution than radiography and can visualize all structures in the knee joint [3,5,35,36]. The degree of tibia anterior translation, which indicates the severity of functional instability, was assessed using an arthrometer [33,37].

The purpose of this study was to compare clinical results and to evaluate the factors associated with cartilage defects in military draftees with ACL rupture among different groups of conservative treatment, post-ACLR with graft rupture, and post-ACLR with intact graft.

## 2. Materials and Methods

This retrospective study was approved by the Institutional Review Board of the Tri-Service General Hospital (approval no.: A202105038).

### 2.1. Population and Study Design

All 126 patients had sustained ACL rupture, and all patients were military draftees who underwent a conscription examination for physical status assessment at the Tri-Service General Hospital in Taiwan from January 2012 to December 2020. The exclusion criteria were multi-ligament knee injury; history of lower limb fracture; history of knee surgery; and history of knee infection.

The patients were divided into three groups:

- Group 1: conservative treatment after ACL rupture.
- Group 2: status post-ACLR, but graft rupture.
- Group 3: status post-ACLR and intact graft.

All patients underwent an MRI scan, thigh circumference measurements, and KT-2000 arthrometer assessment according to the standard military conscription examination.

### 2.2. Measurements

#### 2.2.1. MRI Assessment

MRI was used to evaluate the status of the graft, cartilage defect, and tear of the meniscus in the injured knee. The structural integrity of the graft after ACLR was graded as intact or ruptured according to MRI evaluation [38].

Cartilage defect on MRI of injured knees were classified as present or absent, using the definition of cartilage defects from the MRI Osteoarthritis Knee Score (MOAKS) [39,40].

Meniscal tears were classified as none, lateral meniscus (LM), medial meniscus (MM), or MM + LM tears. All the MRIs were evaluated by experienced radiologists.

#### 2.2.2. Thigh Circumference Difference

Patients were placed in the supine position with the knee extended and were asked to relax their lower extremity. Thigh circumference was measured with tape on both legs, 10 cm above the upper pole of the patella [41,42]. The circumference was measured in millimeters.

The side-to-side difference in thigh circumference represents muscle atrophy between the injured and uninjured sides and is determined by the difference in circumference of the intact side and the injured side.

#### 2.2.3. Knee Joint Anterior Translation Assessment

Anterior translation of the tibia was measured using a KT-2000 arthrometer (MedMetric Co., San Diego, CA, USA), and anterior displacement was measured using the manual maximum force with standardized methods [43,44]. Both knees were measured, and the data are reported in millimeters.

In addition, the side-to-side difference in anterior knee translation between the injured and uninjured sides reliably indicates abnormal knee laxity [45,46].

### 2.3. Statistical Analysis

Data are presented as the mean ± the standard deviation for continuous variables, and the differences between the groups were compared using ANOVA. Categorical variables are expressed as numbers and percentages (%), and differences between groups were compared using the chi-squared test.

To identify the relationship between cartilage defects and variables, the chi-squared test was used for categorical variables, and the independent *t*-test was used for continuous variables. Because the number of some variables was less than 5, Fisher’s exact test was used for adjustment.

Multivariate logistic regression analysis was performed to determine whether age, BMI, thigh circumference difference, side-to-side KT-2000 difference, meniscus tear (LM or MM or LM&MM vs. no tear), and groups (Group 2 or Group 3 vs. Group 1) were factors associated with cartilage defects. The relationships are expressed as odds ratios (ORs) with 95% confidence intervals. The Statistical Package for Social Sciences (SPSS Version 26.0, IBM Corp., Endicott, NY, USA) was used for all statistical analyses. Statistical significance was set at *p* < 0.05.

## 3. Results

### 3.1. Characteristics of the Study

In total, 105 patients were included in this study. Due to the mandatory military service system of Taiwan, all patients in this study were male and relatively young, aged between 22 and 36 years.

All patients were divided into three groups of patients who received conservative treatment after ACL rupture (Group 1, *n* = 40), received ACLR, but graft rupture (Group 2, *n* = 28), and received ACLR with intact graft (Group 3, *n* = 37). Patient demographic data, including age, BMI, and thigh circumference difference, KT-2000 difference, status of meniscal tear, and cartilage defect according to group, are shown in Table 1. The thigh circumference difference (*p* = 0.049) and cartilage defects (*p* = 0.005) were significantly different between the groups.

### 3.2. Relationship with Cartilage Defects

Table 2 demonstrates the relationship analysis of cartilage defects, and significant differences were found between the groups (*p* = 0.005), status of meniscal tear (*p* = 0.014), BMI (*p* = 0.015), and thigh circumference difference (*p* = 0.043).

The multivariable regression model in Table 3 showed that BMI (odds ratio (OR): 1.303; 95% confidence interval: 1.016–1.672; *p* = 0.037), thigh circumference difference (OR: 1.403; 95% CI: 1.003–1.084; *p* = 0.034), LM&MM with no meniscus tear (OR: 13.773; 95% CI: 1.354–140.09; *p* = 0.027), and Group 2 over Group 1 (OR: 5.191; 95% CI: 1.388–19.419; *p* = 0.014) increased the odds of cartilage defect.

No correlation was found between cartilage defect and age, KT-2000 difference, tear of LM over no meniscus tear, tear of MM over no meniscus tear, or Group 3 over Group 1.

## 4. Discussion

The most important finding of our study was that there was a correlation between cartilage defect and BMI, thigh circumference difference, and especially, in post-ACLR, but graft rupture, or status of meniscus tear. Furthermore, patients with ruptured ACL grafts had a higher risk of cartilage defects than those who received conservative treatment.

ACLR was the mainstream opinion in ACL rupture, but there were some studies supporting the conservative treatment, especially for patients with limited demands or older patients [13,14,15,47]. A previous study reported a lower risk of developing degenerative changes with ACL reconstruction, and a delay in reconstruction led to an increasing toll of secondary changes [9]. Another recent study showed no difference in knee OA between operative and non-operative treatment, although knee stability was better in the operative group, but did not result in better functional outcomes [16].

However, complications after ACLR affect clinical performance, and graft failure or rupture may be a major complication. Soderman et al. [3] reported that the incidence of OA of the medial tibiofemoral compartment in ruptured ACL grafts was greater than in intact ACL grafts. A significant increase in cartilage degeneration in the medial femorotibial compartment, followed by the lateral and patellofemoral compartments, was assessed arthroscopically in a study that included 154 patients after failed ACLR at 4 years of follow-up [26]. It was also shown that there was a greater risk of cartilage defects in the graft rupture group than in the intact graft group; even when compared with the conservative treatment group, more cartilage defects were still observed in the graft rupture group. Some previous studies have indicated that graft complications are a well-known devastating event, and there is no doubt that they have more sequelae [3,23,24,25]. However, few studies have compared the differences between graft rupture and non-operation groups.

According to our study, the risk of graft complications should be further assessed when ACLR is considered. This is because once graft rupture occurs, the risk of cartilage defects in the graft rupture group was higher than that in the conservative group. In particular, in patients who had a high risk of graft complications that had increased Beighton scores and greater side-to-side differences in thigh circumference, increased lateral posterior tibial slope, anterolateral tibia subluxation, anteromedial tibia subluxation, and a positive family history of ACL tear [22,26], the necessity of surgery should be carefully considered. For technical errors such as tunnel malposition found during intraoperative or postoperative evaluation [21,22,26], postoperative rehabilitation and protection need to be strengthened, and we must be careful to avoid serious complications such as graft rupture, which will increase the probability of cartilage defects and postoperative OA.

### 4.1. BMI

Some studies have shown that BMI is not associated with early degenerative changes [5] or with primary and revision anterior cruciate ligament reconstruction [48]. However, in our study, BMI revealed a correlation with cartilage defects in all groups with conservative treatment, graft failure, and intact graft.

Recent studies have shown that elevated BMI is highly correlated with graft failure and cartilage degenerative changes after surgery [27]. Snaebjörnsson et al. reported that BMI > 25 and overweight women were risk factors for early ACL revision [29]. Moreover, according to Coggon et al., obesity is a risk factor for surgical failure and OA in meniscus or knee joint problems [49].

Our study further showed that regardless of intact graft or graft rupture after ACLR, conservative treatment after ACL tear and BMI would be considered as risk factors affecting cartilage.

### 4.2. Meniscus Tear

Our results revealed that tears on both sides of the meniscus (LM and MM) increase the complications of cartilage defects. The status of meniscal injury could differ among the conditions of post-ACLR. Meniscal lesions increased from 60% to 86% in all patients between primary and revision ACLR [26]. Another study also reported meniscal tears (medial or lateral) in 94.5% of primary and 83.3% of revision ACLRs and combined (medial and lateral) meniscal tears in 32.1% and 51.1% of primary and revision ACLRs, respectively [22]. In addition, the condition of the meniscus plays an important role in OA progression after ACL injuries. Two-thirds of meniscal injury patients showed OA changes regardless of initial treatment of the ACL injury in the study by Meunier et al. [50], who also reported that the status of menisci was found to be the most important predictor of developing OA. Brophy et al. [27] reported that meniscus disruption was noted in 35% of patients on the medial side and 16% on the lateral side in revision ACLR, and the medial compartment had more severe chondrosis than the lateral compartment.

In contrast with previous studies, our data showed that the greatest influence on cartilage defects was meniscus tear of both sides (odds ratio: 13.773), and no significant difference was observed in tears of the single side (only LM or MM).

### 4.3. Side-to-Side Thigh Circumference Difference

In our study, a statistical difference in side-to-side thigh circumference was found among the groups, suggesting that atrophy in thigh musculature between injured and uninjured limbs might be different. In a study by Otzel et al. [32], 24 ACLR participants were recruited from a university campus, and no significant difference was found in thigh circumference between legs; however, strength deficits were found in the ACLR limb compared to the uninvolved leg. A previous study reported that the thigh circumference of the ACLR limb underestimated thigh atrophy and quadricep weakness persistence, averaging 48 months post-surgery [31].

In contrast, our study showed a statistically positive correlation between bilateral differences in thigh circumference and graft condition after ACLR. Furthermore, quadricep muscle activation and muscle strength are correlated with muscle volume, and quadricep dysfunction is a known risk factor for knee OA [33,51,52] In this study, the greater the side-to-side difference in thigh circumference, the greater the risk of cartilage defects. According to Westlake et al. [53], changes in thigh circumference and mass alter the knee biomechanics during walking and may be related to the risk of knee OA. Particularly in patients who demonstrate hyperlaxity and greater difference in quadricep circumference, this warrants clinical concern [22]. Following rehabilitation guidelines, patients should focus on quadricep strengthening, thigh circumference, and delaying return to sports until quadriceps function has returned.

### 4.4. Side-to-Side Difference of Anterior Knee Laxity with KT-2000

Side-to-side differences in anterior knee laxity in KT-2000 were not significantly different between the groups (Table 1) and did not show a significant influence on cartilage defects (Table 2 and Table 3). Dini et al. [21] reported that the use of the KT-2000 arthrometer showed a statistically significant improvement, and an anterior tibial translation site-to-site difference of ≥3 mm had a significant impact on the Tegner activity scale [33]. However, in our study, the KT-2000 difference showed no significant difference between the groups and had no obvious impact on cartilage defects.

The bones and other non-contractile structures provide joint stability and decrease static stability after ACL injury [54,55]. In clinical practice, the outcome of ACLR is often assessed using tibial translation [56,57], but some studies have shown that tibial translation may not correlate with functional outcomes in patients with ACL deficiency, ACLR, or quadriceps muscle strength [58,59].

### 4.5. ACL and Military Draftees

The study population consisted of military draftees with a history of ACL injury who underwent a conscription examination for physical status assessment.

The rates of OA were significantly higher in military populations than in the general population [60], and a large-scale study of knee OA in an active military population showed that the incidence rates of both primary and secondary OA increased significantly from 2005 to 2014 [11]. Meanwhile, a 10-fold higher incidence of ACL injury has been reported in the military compared to the general population [2], and ACL injury in the military population has a dramatic effect on the ability to continue military duties [10]. Therefore, our findings identified the risk factors of OA after ACLR in military draftees, and careful attention should be paid to those with a history of ACL injury with graft retear, high BMI, tear of both meniscus sides, and a greater side-to-side thigh circumference difference during military training.

The present study had some limitations. First, the study had a retrospective design with a heterogeneous patient cohort, and the small sample size may have affected the statistical significance of the results. Likewise, all patients were male due to being military draftees, and gender differences were not considered [61]. Second, information regarding the condition of the knee joint, such as the meniscus or articular cartilage before ACL injury, and previous injuries to the knee were not available. No data were available on the time interval between the initial knee injury and primary ACLR. Third, we did not quantify the severity of meniscal tears and cartilage defects, which could have influenced clinical outcomes. Finally, this study included patients who underwent surgery by a wide variety of surgeons, which may be a limitation or strength in terms of the generalizability of the findings. Larger-scale and prospective studies should be considered in the future.

## 5. Conclusions

After ACL rupture, the progression of OA is of the most concern. Our study demonstrated that post-ACLR graft rupture, greater thigh circumference differences, BMI, and meniscal tears of both the LM and MM were risk factors for cartilage defects, which represent early degenerative OA changes in the knee.

Thus, with knowledge of these factors, rehabilitation protocols and military training should be customized, depending on individual characteristics, and will, therefore, be more effective for military draftees with ACL injury.

## Figures and Tables

**Table 1:** Patients’ characteristics among groups.

Variables	Group 1 (*n* = 40)	Group 2 (*n* = 28)	Group 3 (*n* = 37)	*p*-Value *
Age, M ± SD	27.8 ± 3.3	26.8 ± 2.7	26.7 ± 3.4	0.298
BMI, M ± SD	24.2 ± 2.5	24.8 ± 2.4	24.6 ± 2.7	0.692
Thigh circumference	13.7 ± 9.0	18.3 ± 14.7	21.4 ± 16.3	0.049
Difference, M ± SD				
KT-2000	3.1 ± 2.7	4.7 ± 4.2	4.9 ± 8.8	
Difference, M ± SD				0.324
Meniscus tear, *n* (%)				
None	8 (20.0%)	3 (10.7%)	10 (27.0%)	0.574
LM	4 (10.0%)	3 (10.7%)	3 (8.1%)	
MM	11 (27.5%)	12 (42.9%)	14 (37.8%)	
LM&MM	17 (42.5%)	10 (35.7%)	10 (27.0%)	
Cartilage defect, *n* (%)				
Present	8 (20.0%)	14 (50.0%)	6 (16.2%)	0.005
Absent	32 (80.0%)	14 (50.0%)	31 (83.8%)	

Data shown as mean ± SD or *n* (%). BMI, body mass index; MM, medial meniscus; LM, lateral meniscus; Group 1: conservative treatment after ACL rupture; Group 2: post-ACLR, but graft rupture; Group 3: post-ACLR and graft intact. * ANOVA or chi-squared test.

**Table 2:** Relationship with cartilage defects.

	Cartilage Defects Present	Cartilage Defects Absent	*p*-Value *
Group, *n* (%)			0.005
1	8 (20.0%)	32 (80.0%)	
2	14 (50.0%)	14 (50.0%)	
3	6 (16.2%)	31 (83.8%)	
Meniscus tear, *n* (%)			0.014
None	1 (4.8%)	20 (95.2%)	
LM	2 (20.0%)	8 (80.0%)	
MM	9 (24.3%)	28 (75.7%)	
LM&MM	16 (43.2%)	21 (56.8%)	
Age, M ± SD	27.5 ± 3.1	27.1 ± 3.3	0.54
BMI, M ± SD	25.5 ± 2.7	24.2 ± 2.4	0.015
Thigh circumference	22.7 ± 15.4	14.2 ± 12.5	0.043
Difference, M ± SD			
KT-2000	4.2 ± 4.3	4.1 ± 6.4	0.923
Difference, M ± SD			

Data shown as mean ± SD or *n* (%). BMI, body mass index; MM, medial meniscus; LM, lateral meniscus; Group 1: conservative treatment after ACL rupture; Group 2: status post-ACLR, but graft rupture; Group 3: status post-ACLR and intact graft. * Chi-squared test or independent *t*-test.

**Table 3:** Logistic regression analysis for cartilage defect.

	β	SE	Odds Ratio (95% CI)	*p*-Value
Age	0.155	0.095	1.168 (0.969–1.406)	0.103
BMI	0.265	0.127	1.303 (1.016–1.672)	0.037
Thigh circumference	0.042	0.02	1.043 (1.003–1.084)	0.034
Difference				
KT-2000	−0.038	0.062	0.963 (0.853–1.087)	0.541
Difference				
Meniscus tear				
LM/None	0.874	1.406	2.396 (0.152–37.724)	0.534
MM/none	1.296	1.182	3.655 (0.361–37.042)	0.273
LM&MM/none	2.623	1.183	13.773 (1.354–140.09)	0.027
Group				
Group 2/Group 1	1.647	0.673	5.191 (1.388–19.419)	0.014
Group 3/Group 1	−0.497	0.768	0.608 (0.135–2.740)	0.517

BMI, body mass index; MM, medial meniscus; LM, lateral meniscus; SE, standard error; β, Regression coefficient; CI, confidence interval. Group 1: conservative treatment after ACL rupture; Group 2: status post-ACLR, but graft rupture; Group 3: status post-ACLR and intact graft.

## Data Availability

All relevant data are within the manuscript files.
